# Efficacy of resuscitative endovascular balloon occlusion of the aorta for hemorrhage control in patients with abnormally invasive placenta: a historical cohort study

**DOI:** 10.1186/s12884-023-05649-8

**Published:** 2023-05-10

**Authors:** Yuanhua Ye, Jing Li, Shiguo Liu, Yang Zhao, Yanhua Wang, Yijing Chu, Wei Peng, Caixia Lu, Chong Liu, Jun Zhou

**Affiliations:** 1 Department of Obstetrics and Gynecology, The Affiliated Hospital of Qingdao University, 16 Jiangsu Road, Qingdao, Shandong Province 266003 China; 2 Prenatal Diagnosis Center, The Affiliated Hospital of Qingdao University, Qingdao, Shandong Province China; 3 Department of Anesthesiology, The Affiliated Hospital of Qingdao University, Qingdao, Shandong Province China; 4 Interventional Medical Center, The Affiliated Hospital of Qingdao University, Qingdao, Shandong Province China

**Keywords:** Cesarean hysterectomy, Obstetric hemorrhage, Placenta accreta spectrum, Abnormally invasive placenta, REBOA

## Abstract

**Background:**

Patients with abnormally invasive placenta (AIP) are at high risk of massive postpartum hemorrhage. Resuscitative endovascular balloon occlusion of the aorta (REBOA), as an adjunct therapeutic strategy for hemostasis, offers the obstetrician an alternative for treating patients with AIP. This study aimed to evaluate the role of REBOA in hemorrhage control in patients with AIP.

**Methods:**

This was a historical cohort study with prospectively collected data between January 2014 to July 2021 at a single tertiary center. According to delivery management, 364 singleton pregnant AIP patients desiring uterus preservation were separated into two groups. The study group (balloon group, n = 278) underwent REBOA during cesarean section, whereas the reference group (n = 86) did not undergo REBOA. Surgical details and maternal outcomes were collected. The primary outcome was estimated blood loss and the rate of uterine preservation.

**Results:**

A total of 278 (76.4%) participants experienced REBOA during cesarean section. The patients in the balloon group had a smaller blood loss during cesarean Sect. (1370.5 [752.0] ml vs. 3536.8 [1383.2] ml; *P* < .001) and had their uterus salvaged more often (264 [95.0%] vs. 23 [26.7%]; *P* < .001). These patients were also less likely to be admitted to the intensive care unit after delivery (168 [60.4%] vs. 67 [77.9%]; *P* = .003) and had a shorter operating time (96.3 [37.6] min vs. 160.6 [45.5] min; *P* < .001). The rate of neonatal intensive care unit admission (176 [63.3%] vs. 52 [60.4%]; *P* = .70) and total maternal medical costs ($4925.4 [1740.7] vs. $5083.2 [1705.1]; *P* = .13) did not differ between the two groups.

**Conclusions:**

As a robust hemorrhage-control technique, REBOA can reduce intraoperative hemorrhage in patients with AIP. The next step is identifying associated risk factors and defining REBOA inclusion criteria to identify the subgroups of AIP patients who may benefit more.

**Supplementary Information:**

The online version contains supplementary material available at 10.1186/s12884-023-05649-8.

## Background

Placenta accreta, or placenta accreta spectrum (PAS), is the direct attachment of the chorionic villi to the uterine wall with partial or complete absence of the decidua [1] and was first described in 1927 [2]. This condition poses the highest risk of life-threatening obstetric hemorrhage if an attempt is made to remove the placenta implanted into the uterine wall forcibly. Recent studies showed that the incidence and prevalence of PAS had separately reached around 1.7 and 37.0, respectively, per 10,000 pregnancies in most high-income countries [3, 4], coincident with the rise in cesarean deliveries worldwide, which is the most common risk factor for PAS.

PAS includes abnormal adherence of the placenta (accreta, when the villi attach to the myometrium) and abnormal invasion of the placenta (AIP), including (increta when the villi invade the myometrium) and percreta (when the villi invade the full thickness of the myometrium) [1]. Compared with accreta, increta, and percreta are more concerning because they are the two main phenotypes of PAS that result in peripartum hysterectomy, maternal morbidity, and even mortality [5].

Cesarean hysterectomy with the placenta in situ is a suggested management method in cases of high suspicion of AIP to reduce the immediate risk of major hemorrhage and accompanying complications [6]. It indicates that a high risk of fertility loss is common among AIP patients. It is undeniably a misfortune for patients who desire future childbirth or those for whom uterus retention is linked to their gender identity and self-esteem [7]. Therefore, such patients should be offered a safe alternative to salvage the uterus while simultaneously removing the implanted placenta.

In order to achieve this goal, a reliable intraoperative hemostasis regimen is imperative, including resuscitative endovascular balloon occlusion of the aorta (REBOA). It is an endovascular hemorrhage control technique successfully used in emergency medicine to maintain blood flow to critical organs in patients with hemorrhagic shock and uncontrolled abdominal, pelvic, or lower extremity bleeding until hemorrhage can be definitively controlled by surgery [8]. REBOA can also be used proactively before the hemodynamic collapse and even before anticipated blood loss, such as obstetric hemorrhage. Therefore, this historical cohort study aimed to evaluate the role of REBOA in managing AIP.

## Methods

### Study population

We retrospectively reviewed the records of patients with singleton AIP pregnancies delivered between January 2014 and July 2021. Due to the retrospective nature of the study, the requirement for informed consent was waived. The inclusion criteria were (1) singleton pregnant patients with placenta increta or percreta diagnosed prenatally using transabdominal or transvaginal ultrasound examination or magnetic resonance imaging (MRI) and confirmed intraoperatively or histologically and (2) a desire to preserve the uterus. Patients with missing values for demographic (e.g., number of previous cesarean delivery) or clinical characteristics (e.g., type of AIP and aortic occlusion time) were excluded.

The prenatal diagnosis of AIP was based on grayscale and color Doppler ultrasound (with bladder filling of 200–300 ml) showing the loss of the “clear zone,” abnormal placental lacunae, bladder wall interruption, myometrial thinning less than 1 mm, placental bulge, focal exophytic mass, uterovesical hypervascularity, subplacental hypervascularity, bridging vessels, or placental lacunae feeder vessels [9]. None of the involved criteria can predict the extent of placental penetration [1], especially for posterior placental implantation and/or obese patients. Therefore, MRI was used to reassess the type of suspected PAS or in cases of equivocal ultrasound findings in at-risk patients. Intraoperatively, placenta increta or percreta was reconfirmed in patients with chorionic villi invading the myometrium or perimetrium (uterine serosa). Surgical samples (including separated placentas, total or partial hysterectomy tissues, and partially resected myometrium with or without implanted placenta) were obtained whenever possible for histopathological confirmation.

**Fig. 1:**
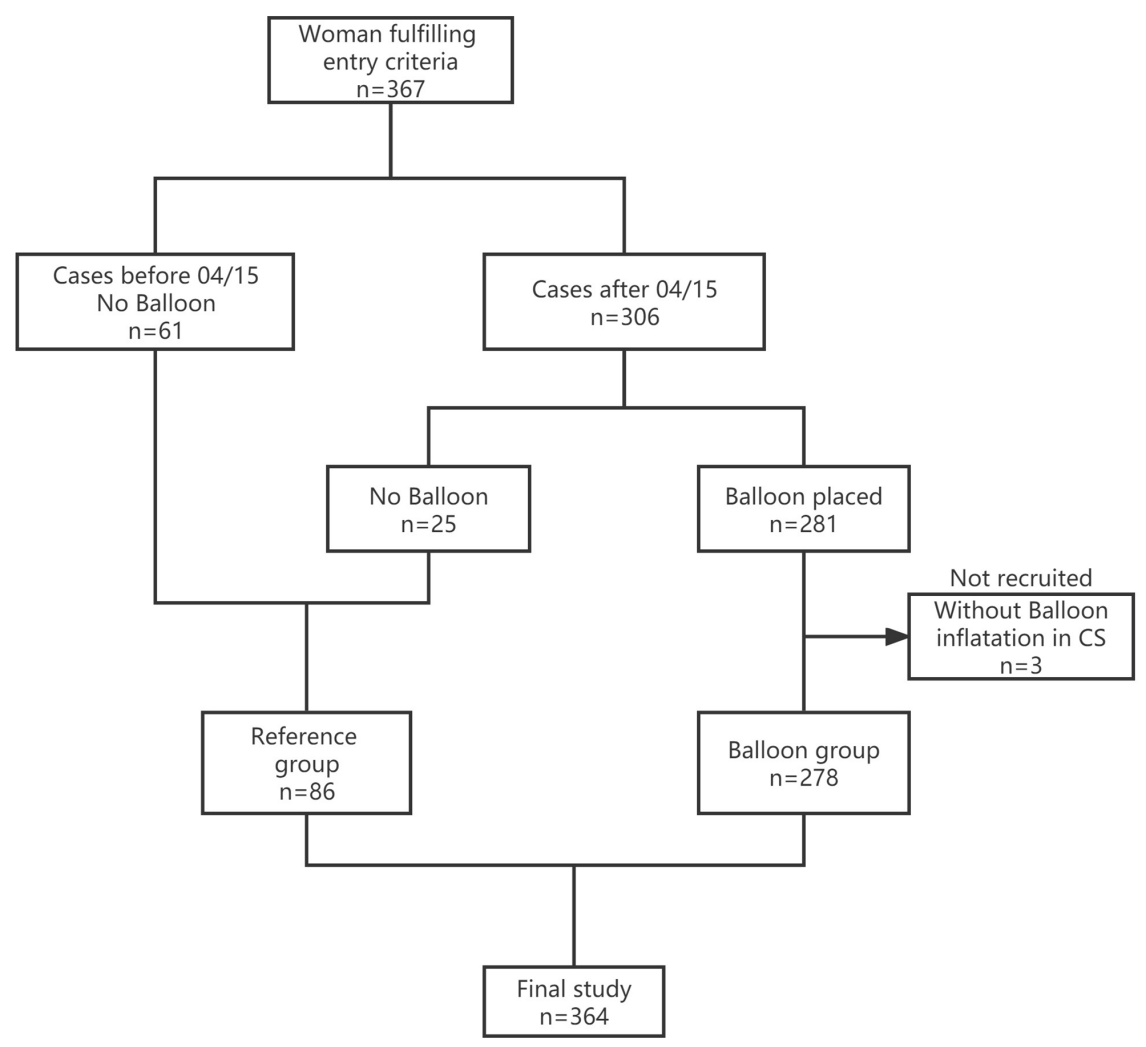
Flow chart of study population selection

All patients were allocated to two groups: those who underwent REBOA during cesarean section (balloon group, n = 278) and those who did not undergo REBOA (reference group, n = 86). Most patients (61/86) in the reference group underwent cesarean section without aortic balloon before April 2015, when the REBOA technique was first performed in AIP patients in our tertiary referral hospital. After April 2015, REBOA was not performed in the remaining patients (25/86) in the reference group because the interventional radiology service was unavailable before their cesarean sections. A total of 281 patients underwent lower abdominal aortic balloons before cesarean section; however, three balloons were not inflated during surgeries at the surgeon’s discretion because their placental invasion was not as severe as initially thought. Excluding these patients, 278 remain in the balloon group (Fig. 1). Only patients with planned cesarean sections were included to ensure the availability of the REBOA team.

### Management protocol

After admission, written informed consent was obtained from all patients, thoroughly acknowledging their clinical condition and the latent risks and uncertain benefits of various treatments. The delivery date was determined by multidisciplinary counseling for each case based on ultrasound and MRI results, prenatal vaginal bleeding, uterine contractions, and fetal maturity.

Before cesarean section, the patients in the balloon group were sent to the interventional radiology surgical suite. The right femoral arterial puncture was performed under local anesthesia by an interventional radiologist using a 12.0-F occlusion balloon catheter (MAXI-LD 20⋅40 110CM REF: 4,162,040 L, Cordis Corp., Miami Lakes, FL, USA) with a 0.035-in stiff guidewire (THSCF-35-145-1.5-ROSEN; Cook) placed using a 12.0-F introducer sheath (Ultimum EV Hemostasis; St. Jude Medical., Minnetonka, MN, USA). The balloon was accurately placed under the renal artery level at the distal abdominal aorta and above the bifurcation of the abdominal aorta under fluoroscopic guidance. Simultaneously, using trial balloon inflation, the accurate placement of the balloon was confirmed, as well as the exact volume of 0.9% saline needed to be injected into the balloon to obtain the ideal vascular occlusion (Videos S1 and S2). Subsequently, the patients were transferred from the interventional radiology suite to the operative theater for cesarean section under general anesthesia.

**Fig. 2:**
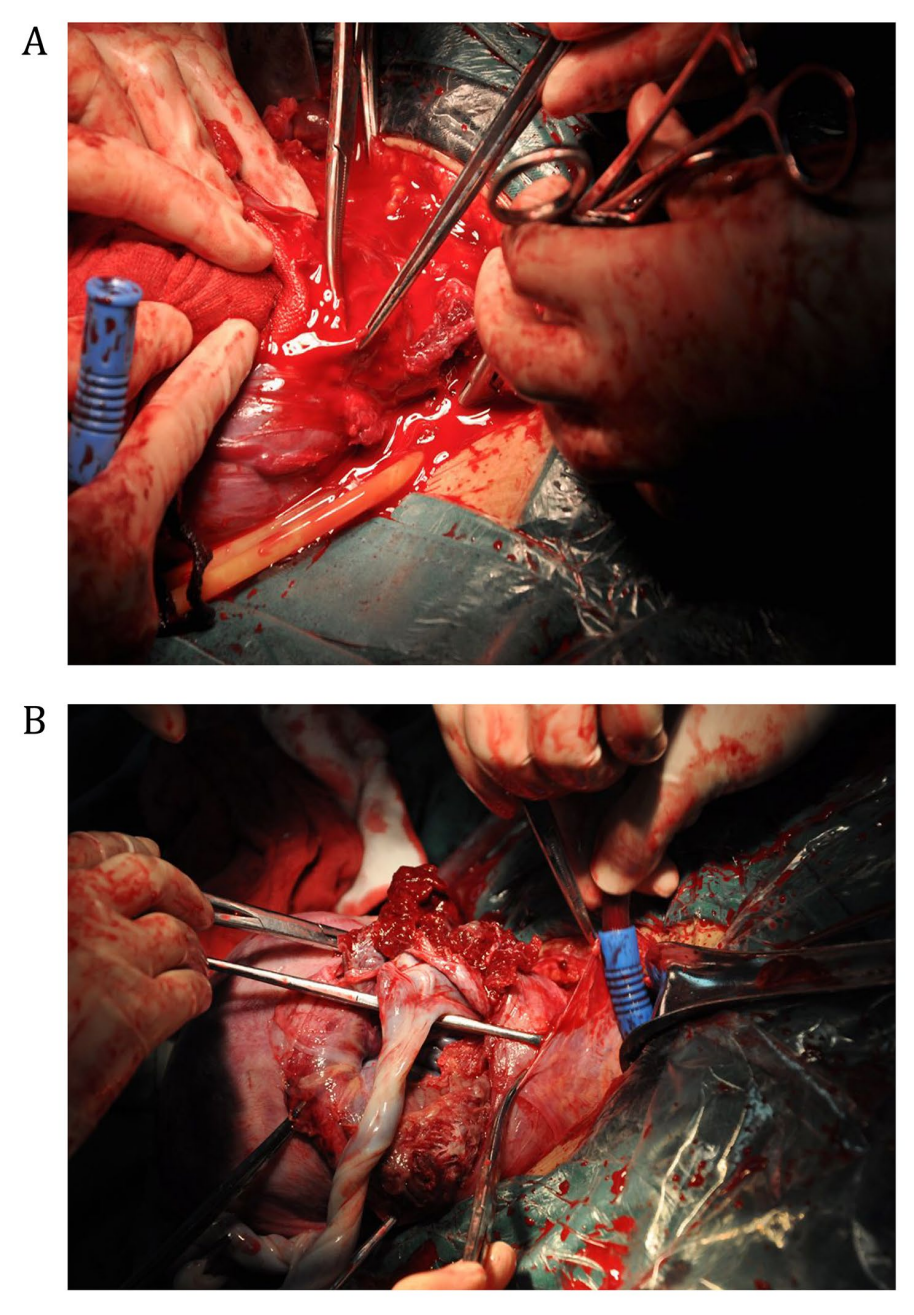
Parallel traversal compressing suture technique. (**A**) This novel parallel traversal compressing suture technique was used to strengthen the weak uterine lower segment without contractile force after placental dissection, and the corresponding blood vessels were ligated for hemostasis. (**B**) Diagrammatic sketch of parallel traversal compressing suture technique

**Fig. 3:**
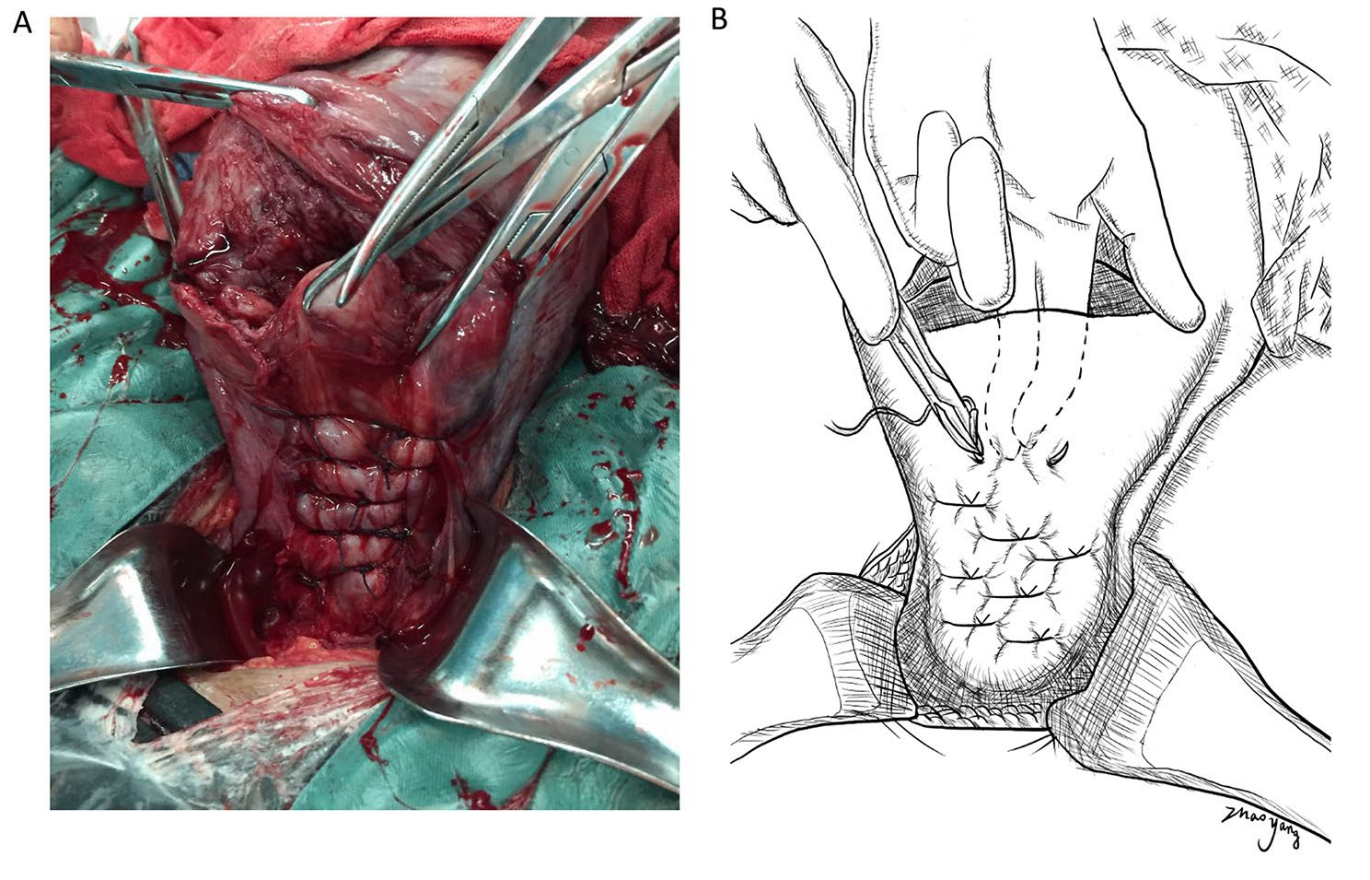
AIP surgical field with or without REBOA. (**A**) Performing cesarean hysterectomy in a bloody operating field without REBOA. (**B**) Inflation of the aortic balloon after umbilical cord clamping to provide a clear surgical field. REBOA, resuscitative endovascular balloon occlusion of the aorta

A multidisciplinary team performed all cesarean sections. The REBOA procedures were performed under Doppler fetal heart monitoring to diagnose fetal distress. General anesthesia and acute placenta-related hemorrhage increase the suffocation and other complications of the perinatal fetuses. Neonatal pediatricians were present at the time of delivery. Neonates were evaluated after delivery, transferred to NICU when necessary, and other neonates returned to obstetrics. Hysterectomy was performed if the operating team confirmed that preserving the uterus was unfeasible. Bilateral ureteral stents were inserted before or during the operation if necessary. In the reference group, following fetal delivery, a tourniquet was usually used to tie around the cervix to help reduce bleeding. Usually, there was no clear surgical field because of excessive bleeding (Fig. 2A). On the contrary, in the balloon group, in the case of fetal delivery and cord clamping, the aortic balloon was inflated with 0.9% saline to reduce blood supply to the operating field to obtain a cleaner operating field (Fig. 2B). Before trying to remove the placenta, the bladder flap was dissected thoroughly from the lower anterior uterine surface (surpassing the internal os level). Following removing the placenta, a parallel traversal compressing suture technique (four knots: while ensuring the fixation of the suture, complete the compression suture as quickly as possible to shorten the blocking time and related complications) (Fig. 3) was applied to stop bleeding, ligate the blood vessels, and remodel the uterus.

During surgery, every 10–15 min of occlusion of the aorta, there would be a 1-min deflating time of the balloon to resuscitate ischemic organs until ideal hemostasis was observed. It was performed until the balloon was deflated and removed. After the operation, the sheath and occlusion balloon catheter would be removed 4–6 h later if vital signs were stable and lower extremity perfusion was assessed before, during, and after balloon occlusion and after sheath removal. Otherwise, once continuous uterine bleeding was confirmed, bilateral uterine artery embolization or hysterectomy would be performed depending on the situation, e.g., non-life-threatening or uncontrolled bleeding. Low-molecular-weight heparin was prophylactically administered to all patients in both groups 12 h after surgery to reduce the risk of venous thromboembolism.

The following parameters were recorded: demographic data, estimated blood loss, transfusion of packed red blood cells, operation duration, hysterectomy performed, bilateral uterine artery embolization required, aortic occlusion time, femoral artery cannulation time (time from the percutaneous femoral artery puncture to successful placement of the introducer sheath), indwelling sheath duration (femoral artery cannulation time), radiographic exposure (mGy) and screening time, total maternal care costs, intensive care unit (ICU) and neonatal ICU (NICU) admission, and AIP surgery- and balloon catheter-related complications.

ICU monitoring after the operation was necessary, and attention was paid to the pulse of dorsalis pedis artery for early detection of lower extremity arterial thrombosis. Usually, an ultrasound of the lower limb vessel was performed 1 day after surgery. If vaginal bleeding permitted, low-molecular-weight heparin (LMWH) was 12 h after surgery to avoid thrombosis. Vaginal bleeding and multiple internal environmental hemostases were monitored.

### Statistical analysis

For continuous variables, the Kolmogorov-Smirnov test was used to test the normal distribution of the data. The continuous variables that conformed to the normal distribution were shown as means (SD) and analyzed using Student’s t-test; those that did not conform to the normal distribution were expressed as medians (ranges) and were analyzed using the Mann-Whitney U-test. Categorical variables were expressed as n (%) and analyzed using Pearson’s chi-squared test or Fisher’s exact test, as appropriate. In order to define the risk factors of thrombosis, univariable logistic regression was performed, and the results were expressed as odds ratios (Ors) with their 95% confidence intervals (CIs). IBM SPSS Statistics for Mac version 25.0 (IBM Corp., Armonk, NY, USA) was used for all analyses. *P*-values < 0.05 were considered statistically significant.

## Results

### Patient demographics

No significant differences in maternal age, gravidity, parity, number of previous cesarean deliveries, grade of placenta previa (minor or major), and type of AIP were observed between groups (Table 1). However, there was significant difference in gestational age at delivery between two groups (*P* = .01).

**Table 1:** Demographic and obstetric characteristics of patients

	Balloon group (*n* = 278)	Reference group (*n* = 86)	*p*-value
Maternal age, years	34.00(30.00–37.00)	34.00(32.00–36.00)	0.84
Gestational age at delivery, weeks	36.20(35.00-37.20)	36.60(36.00-37.30)	0.01
Gravidity	4.00(3.00–5.00)	4.00(3.00–5.00)	0.25
Parity	1.00(1.00–1.00)	1.00(1.00–2.00)	0.06
Number of previous cesarean deliveries	1 (0–3)	1 (0–2)	0.45
Preoperative bleeding	81 (29.1)	23 (26.7)	0.78
Combined placenta previa			0.49
Major	207 (74.4)	65 (75.6)	
Minor	59 (21.2)	15 (17.4)	
None	12 (4.3)	6 (7.0)	
Type of AIP			0.23
Increta	203 (73.0)	57 (66.3)	
Percreta	75 (27.0)	29 (33.7)	
Neonatal outcomes			
Birthweight, mean (SD), g	2805 (290)	2890 (323)	0.49
NICU admission	176 (63.3%)	52 (60.4%)	0.70
1-min APGAR score, mean (SD)	8.87 (1.15)	8.58 (1.41)	0.26
5-min APGAR score, mean (SD)	9.57 (0.81)	9.35 (1.36)	0.36

Data are given as mean (SD)n (%) or median (range)

*P* < .05 was considered statistically significant

### Comparisons of patient outcomes

In the balloon group, all patients successfully underwent occlusion balloon placement before delivery, and all balloons were well inflated following cesarean section. The median time taken for attempted femoral artery cannulation was 130 (range, 75-1265) s. The mean (SD) fluoroscopy time was 2.8 [1.3] (range, 1.4–5.6) s. The mean estimated maternal radiation exposure was 2.3 [1.0] (range, 1.2–4.6) mGy, which is considered safe in pregnancy [10].

**Fig. 4:**
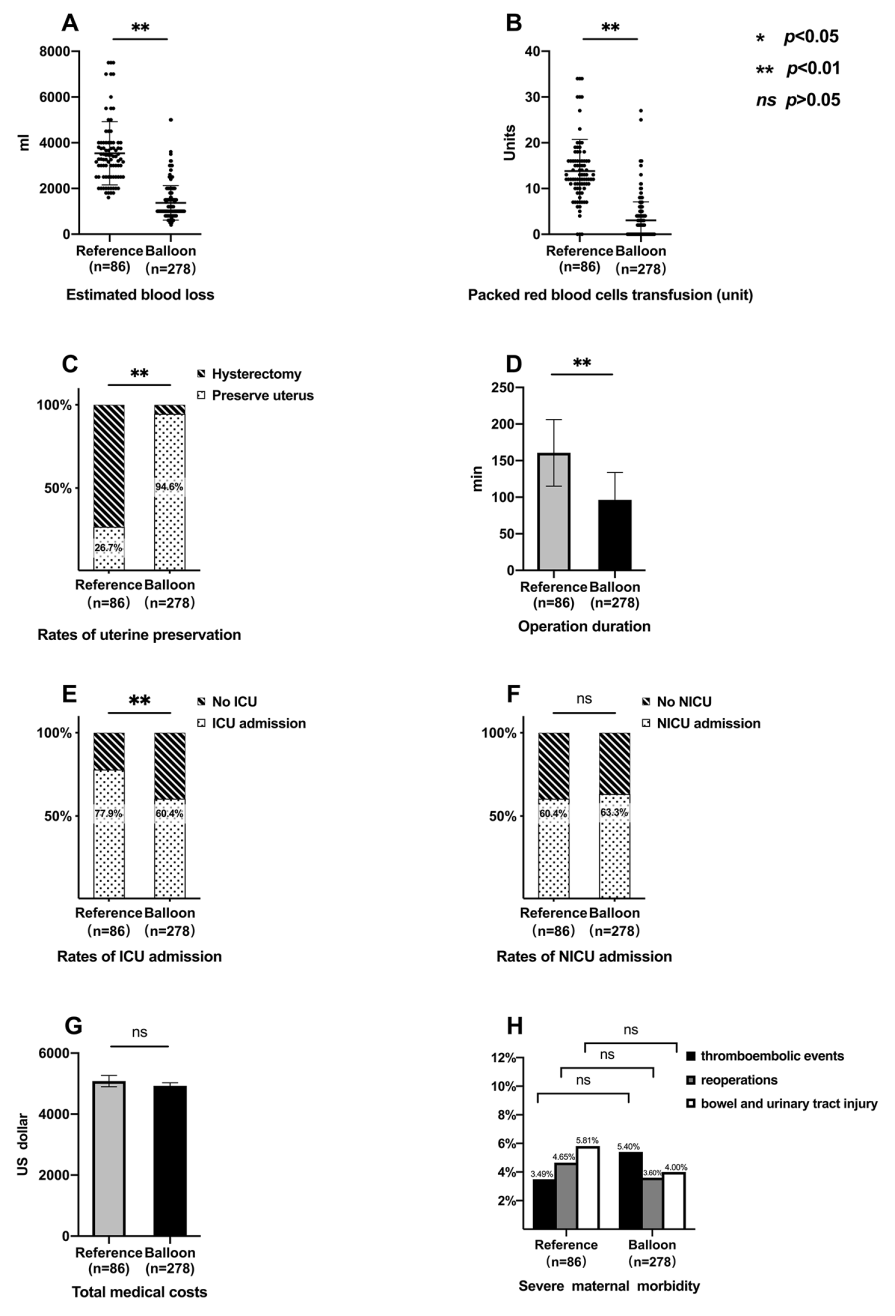
**Comparisons of patient outcomes between the reference and balloon groups** (**A**) Intraoperative estimated blood loss in the balloon group compared with the reference group; *P* < .001, independent-sample t-test. (**B**) Comparison of PRBC transfusions between the balloon and reference groups. *P* < .001, Mann–Whitney U test. (**C**) Rate of uterine preservation in the balloon group compared with that in the reference group; *P* < .001, Chi-squared test. (**D**) Comparison of operation duration between the balloon and reference groups; *P* < .001, independent-samples t-test. (**E**) Rate of ICU admission in the balloon group compared with that in the reference group; *P* = .003, Chi-squared test. (**F**) Rate of NICU admission in the balloon group compared with that in the reference group; *P* = .70, Chi-squared test. (**G**) Comparison of total medical costs between the balloon and reference groups; *P* = .13, independent-samples t-test. (**H**) Rates of thromboembolic events, reoperations, and bowel or urinary tract injury in the balloon group compared with that in the reference group; *P* = .707, *P* > .999, *P* = .399, respectively, Fisher’s exact test. ICU, intensive care unit; NICU, neonatal intensive care unit

The mean (SD) estimated blood loss during surgery were 1370.5.4 [752.0] ml and 3536.8 [1383.2] ml in the balloon and reference groups, respectively (*P* < .001) (Fig. 4A). Accordingly, fewer units of packed red blood cells were transfused in the balloon group than in the reference group (3.0 [4.0] vs. 13.8 [6.9], *P* < .001, respectively) (Fig. 4B). There were 264/278 (95.0%) and 23/86 (26.7%) patients preserving their uterus successfully in the balloon and reference groups, respectively (*P* < .001) (Fig. 4C). The mean (SD) operation duration in the balloon group was significantly shorter than that in the reference group (96.3 [37.6] min vs. 160.6 [45.5] min) (*P* < .001) (Fig. 4D). The patients in the balloon group had a lower ICU admission rate (168/278, 60.4%) than their counterparts in the reference group (67/86, 77.9%, *P* = .003) (Fig. 2E). The NICU admission rate showed no significant differences between groups (176/278 (63.3%) vs. 52/86 (60.4%), *P* = .70) (Fig. 4F). No differences in mean (SD) total maternal medical costs were found between groups ($4925.4 [1740.7] vs. $5083.2 [1705.1], *P* = .13) (Fig. 4G).

### Surgery- and catheterization-related complications

There were no cases of sepsis, peritonitis, fistula, acute pulmonary edema, acute renal failure, pulmonary embolism, or death in either group. For other severe morbidities, including reoperations (repeat laparotomy to perform hysterectomy or hemostasis, abdominal wall hematoma removal, uterine artery embolization, and thromboembolectomy) and thromboembolic events (deep vein thrombosis and arterial thrombosis), bowel or urinary tract injury also showed no significant differences between groups (Fig. 4H). A summary of the severe maternal morbidities is provided in Table 2. In the balloon group, four of eight patients who had femoral arterial thrombosis underwent thromboembolectomy. The remaining four patients were successfully managed with thrombolytic therapy. The frequency of thrombolysis and thromboembolectomy had an impact on the balloon procedures. The main symptoms of ureteral injury after surgery (n = 11, 3.96%) were lumbar discomfort, decreased urine volume, and increased leucocyte count. Then, the unilateral ureter and hydronephrosis were found by color ultrasound of the urinary system, which could help diagnose the ureteral injury. Most cases underwent insertion of double-J with an improvement of the renal function.

**Table 2:** Cases of severe maternal morbidity in reference and balloon group

Severe maternal morbidity	Reference group	Balloon group	*p*-value
**Reoperations**			
Abdominal wall hematoma removal	3 (3.5%)	3 (1.1%)	
laparotomy with hysterectomy	1 (1.2%)	3 (1.1%)	
Thromboembolectomy	0	4 (1.4%)	
**Thromboembolic events**			
Deep vein thrombosis	3 (3.5%)	7 (2.5%)	
Arterial thrombosis	0	8 (2.9%)	
**Injury to adjacent organs**			
Bowel injury	1 (1.2%)	0	
Urinary tract injury	4 (4.7%)	11 (4.0%)	
Total (%)	14.0 (12/86)	12.9 (36/278)	0.81

In the balloon group, the mean (SD) occlusion time was 14.6 [8.3] (range, 5–54) min. Overall, 7.19% (20/278) of the patients experienced interventional radiology-related complications. Of the 278 patients, regular uterine contractions after the intervention was detected in 12 (4.32%), local catheter-related infections in two (0.72%), local hemorrhage in three (1.08%), and hematomas in three (1.08%) patients.

### Risk factors for thrombosis

Based on clinical experience, aortic occlusion time, estimated blood loss, operation duration, femoral artery cannulation time, and indwelling sheath duration were selected as possible variables associated with femoral arterial thrombosis. In the univariable logistic regression analysis (Table 3), none of these variables were associated with an increased thrombotic risk at the femoral site.

**Table 3:** Predictors of thrombotic risk in the femoral location in the balloon group

Characteristics	Femoral arterial thrombosis		Unadjusted OR (95% CI)	*p*-value
	Yes, *n* = 8	No, *n* = 270		
Aortic occlusion time (m)	13 (10–30)	12 (5–54)	1.06 (0.95–1.18)	0.30
Estimated blood loss (ml)	1200 (500–3500)	1000 (400–5000)	0.99 (0.98–1.00)	0.09
Operation duration (m)	68 (60–235)	90 (42–285)	1.01 (0.98–1.03)	0.59
Femoral artery cannulation time (s)	140 (90–775)	130 (75–1265)	0.98 (0.95–1.00)	0.21
Indwelling sheath duration(h)	7.5 (4.0–26.0)	7.5 (4.5–30.0)	0.99 (0.95–1.00)	0.60

**Abbreviations:** CI, confidence interval; OR, odds ratio. Continuous variables are presented as median (range)

## Discussion

Obstetric hemorrhage is the principal cause of maternal morbidity and mortality worldwide [11]. AIP is one of the most likely obstetrical factors to cause massive postpartum hemorrhage. Therefore, managing AIP by effectively reducing blood loss and associated maternal morbidity and mortality rates is essential. The present study excluded patients with abnormal adherent placenta, which might confound the role of REBOA in managing AIP. This study reported decreased intraoperative hemorrhage and a lower need for hysterectomy for severe PAS disorders with the prophylactic use of REBOA.

REBOA was not performed in all patients who wished uterine preservation. Regardless of whether the patient intended to retain the uterus or not, the patients were first informed of the benefits and risks of balloon blocking and then performed REBOA after informed consent. Therefore, all enrolled patients wished to retain their uterus. With the assistance of balloon occlusion, a clearer surgical field and operation opportunity could be obtained. The operator could judge whether an attempt to retain the uterus could be performed in a patient with increta or percreta. The REBOA options were provided for patients with placenta increta and percreta in the preoperative evaluation. Otherwise, once continuous uterine bleeding was confirmed, bilateral uterine artery embolization or hysterectomy would be performed depending on the situation, e.g., non-life-threatening vs. uncontrolled bleeding. If the area of placenta implantation was wide and the operator expected that the wound formed by the lower segment of the uterus after placenta stripping would is too large to repair or reconstruct, then a hysterectomy was directly performed.

After the fetus was delivered, the operating field’s blood supply was blocked by the inflating abdominal aortic balloon, and a relatively “dry” and clean surgical field could be obtained. It allowed the surgeon to proceed quickly and accurately with bladder dissection, remove the implanted placental tissue, and quickly perform compression hemostatic sutures or repair the extremely thin uterine wall where the placenta was detached. It can help shorten the duration of surgery.

Although ultrasonography combined with MRI has shown satisfactory sensitivity and specificity in the prenatal diagnosis of AIP [12], three patients whose intraoperative findings were incompatible with the prenatal findings were excluded. The balloon was not inflated during the operation in these three patients. From this point, the balloon was prophylactically placed, rather than prophylactically inflated, in patients with a higher suspicion of AIP to avoid unnecessary risks associated with aortic occlusion because of false-positive prenatal diagnoses.

Unlike REBOA used in trauma and emergency medicine, the prophylactic placement of an endovascular balloon occlusion catheter can be a fundamental component of surgical AIP management. Indeed, REBOA can be deployed in pregnant women with established hemorrhagic shock during an emergency peripartum hysterectomy as a rescue maneuver to maintain the stability of the circulatory system [13].

The safety and efficacy of REBOA in women with a prenatal diagnosis of AIP are yet to be confirmed [14]. Published results are conflicting, ranging from affirmative [15] to skeptical [16] to inconclusive [17]. Nevertheless, most studies involved small numbers of patients or did not distinguish abnormal adherent placenta (i.e., accreta) from AIP (i.e., increta and percreta), despite AIP being the main cause of massive obstetric hemorrhage and infertility [18]. Given the lack of effective hemostasis, even handled by an experienced multidisciplinary team of surgeons, peripartum hysterectomy presents considerable risks because of possible pelvic adhesions from previous surgery, the enlarged blood supply to the pregnant uterus, and possible invasion to the parametrium in cases of placenta percreta. Therefore, the morbidity and mortality associated with surgery for AIP are significant, and perioperative complications are common [4, 19]. REBOA provides time and opportunity for surgeons to perform surgery deliberately, reduce the complexity of the operation, and eventually lower the technical challenge of managing AIP. A cleaner operating field is a prerequisite for properly laying out the compressive sutures. Compressive sutures without REBOA can be impossible because of the massive and rapid hemorrhage. Nevertheless, parallel compression suture was reported as a new suturing method for hemostasis from the lower uterine segment in women with placenta previa and accrete [20]. Other compression suture methods were reported to control placenta previa and accrete, such as longitudinal or vertical parallel compression, but they were not used here. There were no significant differences in the urinary tract or bowel injury rates between the groups. It may be because all patients were managed by an experienced multidisciplinary care team, and each group had a small number of cases of complications.

REBOA deployment-related complications are another concern, including those related to femoral artery cannulation, balloon positioning and removal, and thromboembolic events. Excluding mild complications, arterial thrombosis is one of the most common severe complications of REBOA, with a reported incidence of 5% [21]. Lower limb ischemia, pseudo-aneurysm formation, ischemic femoral nerve injury, and maternal death due to abdominal aortic dissection have been reported [22]. Obstetric hemorrhage offers expanding indications for REBOA, and each complication’s exact incidence and predictors remain unclear. In this study, there were 8/278 (2.88%) cases of arterial thrombosis in the balloon group, but no vascular injuries or lower limb ischemic episodes. It appears that percutaneous cannulation disrupts the integrity of the vessel wall, triggering thrombus formation. Women generally have small-caliber arteries, which is independently associated with arterial obstruction [23], and pregnancy is a risk factor for hypercoagulability. These patients may be more vulnerable to thrombosis because of the administration of tranexamic acid or other coagulation factors. In order to determine the risk factors for arterial thrombosis, univariable logistic regression analysis was performed in the present study, but no associated variables were found, despite some studies have reported that longer catheter dwell times, prolonged femoral artery cannulation times, and multiple punctures for catheter insertion are associated with thrombosis or embolism [24]. The small number of arterial thrombosis cases may be a reason; therefore, multicenter prospective studies are needed to confirm predictors of thrombotic risk of REBOA in obstetrics. Although long occlusion time (4–6 h) of the infrarenal aorta is possible [25], the strategy was intermittent deflation of the balloon to relieve ischemia and removing the catheter and sheath as soon as possible while the vital signs remain stable. As anticipated, common femoral artery cannulation performed by skilled hands and smaller introducer sheaths for REBOA might be associated with fewer access-related complications [26].

Considering REBOA-related complications, most findings were generated from their application in trauma patients with hemorrhagic shock. Although the small number of cases in the present study prevents definite conclusions, the patients should be informed of the risk of arterial thrombosis. Moreover, AIP patients should be counseled on the increased likelihood of recurrence in subsequent pregnancies before choosing conservative treatment, for the recurrence among women with PAS who retained their uterus is 13.3-29.6% [27, 28]. The next step should be identifying associated risk factors and defining REBOA inclusion criteria to identify the subgroups of AIP patients who benefit more from REBOA.

In this study, 14 patients in the balloon group failed to retain their uteri. Even with the support of REBOA, it remains challenging to prejudge the chances of preserving the uterus successfully at the point when the peritoneum is opened. The REBOA-assisted cesarean section remains a novel method for AIP patients, and the intraoperative strategy and patient outcomes also need to be improved over time.

Including expenditures related to vascular and interventional radiology processes, the total medical costs in the balloon group showed no significant differences from those in the reference group. Undoubtedly, the decreased blood product requirement lowered the total medical care costs. The lower need for blood transfusion and ICU admission in the balloon group means that REBOA is particularly suitable for regions where blood products are relatively restrained.

An alternative interventional radiologic modality used in women with PAS is the occlusion of the internal iliac arteries. Nevertheless, this process appears more debatable regarding its benefits regarding hemostasis than those associated with aortic occlusion [29, 30]. The extensive collateral blood supply of the pelvis most likely explains it. Randomized controlled studies are needed to examine the risks and benefits of REBOA compared with those of internal iliac artery occlusion. What is certain is that REBOA only requires cannulation of a single common femoral artery, and the exposure dose to the fetus is considered lower and is, therefore, acceptable [10].

This study has some limitations. This study had a single-center and retrospective design. Moreover, follow-up data on future pregnancies following this intervention were not available. Without knowledge of how these patients do in future pregnancies, the role of REBOA in preserving fertility in AIP patients cannot be revealed. In addition, the skills of the REBOA operator were not evaluated. A future multicenter prospective study is needed to expand on this innovative approach. Furthermore, the cost-effectiveness analysis is likely only applicable to China. Finally, and perhaps most importantly, hysterectomy without removal of the placenta is strongly recommended in patients with percreta [5, 6, 14, 18], but such an option is unacceptable to many Chinese women who wish to retain fertility and if other options are available. It is why the uterus-sparing strategy is being sought. Other healthcare systems may or may not see a benefit.

## Conclusions

This study demonstrated that REBOA, as an adjunct therapeutic strategy for hemostasis, can help reduce intraoperative blood loss and salvage the uterus in AIP patients.

## Electronic supplementary material

Below is the link to the electronic supplementary material.

Additional file 1

Additional file 2

Additional file 3

## Data Availability

All data generated or analyzed during this study are included in this published article and its supplementary information files.

## Abbreviations

AIP: Abnormally invasive placenta
PAS: Placenta accreta spectrum
REBOA: Resuscitative endovascular balloon occlusion of the aorta
ICU: Intensive care unit
NICU: Neonatal intensive care unit
MRI: Magnetic resonance imaging
CI: Confidence interval
OR: Odds ratio
